# Knoxville City Dental Society

**Published:** 1892-11

**Authors:** 


### Knoxville City Dental Society.

The Dentists of Knoxville, Tenn., have organized a city association to meet monthly. Its objects, rules and regulations are about the same as those of other local societies, viz: for maintaining the status of the profession, promoting its best interests, and for mutual professional and social benefit.

The officers are as follows: Pres. J. T. Cazier; Vice Pres. R. N. Kesterson; Treasurer, A. R. Melenda; Secretary, A. J. Cottrell.

The Committee of Arrangement: W. B. Robinson, and B. D. Brabson.

This is certainly a move in the right direction, and it will result in much good to the profession and the public in Knoxville and its vicinity.

The "Register" will gladly publish reports of proceedings and papers read in the society.

